# Diet Quality Limits Summer Growth of Field Vole Populations

**DOI:** 10.1371/journal.pone.0091113

**Published:** 2014-03-12

**Authors:** Kristian M. Forbes, Peter Stuart, Tapio Mappes, Katrine S. Hoset, Heikki Henttonen, Otso Huitu

**Affiliations:** 1 Suonenjoki Research Unit, Finnish Forest Research Institute, Suonenjoki, Finland; 2 Department of Biological and Environmental Science, University of Jyväskylä, Jyväskylä, Finland; 3 Department of Botany and Zoology, Masaryk University, Brno, Czech Republic; 4 Section of Ecology, Department of Biology, University of Turku, Turku, Finland; 5 Vantaa Research Unit, Finnish Forest Research Institute, Vantaa, Finland; CNRS, Université de Bourgogne, France

## Abstract

Marked variation occurs in both seasonal and multiannual population density peaks of northern European small mammal species, including voles. The availability of dietary proteins is a key factor limiting the population growth of herbivore species. The objective of this study is to investigate the degree to which protein availability influences the growth of increasing vole populations. We hypothesise that the summer growth of folivorous vole populations is positively associated with dietary protein availability. A field experiment was conducted over a summer reproductive period in 18 vegetated enclosures. Populations of field voles (*Microtus agrestis*) were randomised amongst three treatment groups: 1) food supplementation with *ad libitum* high protein (30% dry weight) pellets, 2) food supplementation with *ad libitum* low protein (1% dry weight; both supplemented foods had equivalent energy content) pellets, and 3) control (no food supplementation), n = 6 per treatment. Vole density, survival, demographic attributes and condition indicators were monitored with live-trapping and blood sampling. Highest final vole densities were attained in populations that received high protein supplementation and lowest in low protein populations. Control populations displayed intermediate densities. The survival rate of voles was similar in all treatment groups. The proportion of females, and of those that were pregnant or lactating, was highest in the high protein supplemented populations. This suggests that variation in reproductive, rather than survival rates of voles, accounted for density differences between the treatment groups. We found no clear association between population demography and individual physiological condition. Our results demonstrate that dietary protein availability limits vole population growth during the summer growing season. This suggests that the nutritional quality of forage may be an underestimated source of interannual variation in the density and growth rates of widely fluctuating populations of herbivorous small mammals.

## Introduction

Populations of northern small mammals are renowned for their high-amplitude density cycles, with peaks every 3–5 years [1]–[5]. Although delayed density-dependent predation is often considered the principle driver of cyclic dynamics [6]–[10], regulatory processes are likely to be multifactorial and geographically variable [1], [11]. Hence, consensus on causal factors behind cyclicity has not been reached despite several decades of research [1], [10]–[14].

Boreal vole cycles typically involve two successive years of variable but positive population growth in summer and negative or zero population growth in winter [2], [15]–[16]. The peak of a multiannual cycle is usually attained in late summer to autumn of the latter increase year, after which winter food depletion initiates a population decline [17]–[18]. The year following peak density is characterized by a summer decline, when populations typically decrease in size from spring to autumn [1]–[2], [15].

The growth rate of vole populations varies profoundly between years, including years representing the same cycle phase. The overall amplitude of multi-annual cycles (i.e. the difference between maximum and minimum densities) also varies markedly within and between sites [1]–[2], [8], [19]. Cycle amplitude is generally greater in cooler and more continental areas than in temperate, mild coastal areas, where density variations are predominantly seasonal [1], [20]. These differences have traditionally been attributed to winter severity and amount of snowfall, which are negatively associated with the stabilising effect of generalist predators on voles and their specialist predators [1], [8].

Recent reports have documented a widespread collapse of small rodent population cycles [21], often attributed to changing winter climate [22]–[24]. Korpela et al. [19] presented evidence to challenge this association, and instead, using extensive time-series vole monitoring and climatic data from Finland, highlighted a connection between weather conditions during spring and summer, and vole population growth. The latter relationship is potentially mediated by variation in forage quality (e.g. [25]–[26]).

For herbivores, forage quality is to a considerable degree determined by nitrogen content, which is often a primary limiting factor for the growth of populations (nitrogen limitation hypothesis [27]–[30]). Nitrogen levels in plants vary in response to a range of biotic and abiotic factors, such as weather, leading to both spatial and temporal variation in its availability to herbivores [31]–[32]. For example, Cole and Batzli [33] identified that different vegetation types altered the density, reproductive performance and survival of wild prairie vole populations, and concluded that highly nutritional forage can elevate peak population densities. Additionally, a midsummer cessation of breeding, often occurring in cyclic folivorous voles during the height of the summer growing season (termed a ‘midsummer crisis’ for voles), is hypothesized to result from nutritional changes in plants during their reproductive phenology [15].

The physiological health state of individuals may vary before translating into changes in population demography. For example, populations of small mammals are characterised in decline years by small individual body size, as well as low reproductive output and adult survival (the Chitty effect [34]–[36]). Haematological indices, e.g., levels of albumin, haematocrit and immunoglobulins, can also reflect the quality of individual dietary intake [37]–[40].

The objective of our study is to evaluate the extent to which protein availability limits the density and population growth of small mammals during northern European summer, a time of seemingly superabundant food resources. We hypothesise that protein supplementation will have positive effects, proximately on the physiological condition of voles and ultimately on population growth, as compared to non-supplemented populations. Specifically, we predict that the positive response will be more pronounced in populations that receive high protein food than in those that receive supplemental food with equivalent levels of energy but low protein. As model species we use the folivorous field vole (*Microtus agrestis*), the most widely distributed of fluctuating small rodents throughout Fennoscandia, and often considered the driver of population cycles in northern Europe [41].

## Materials and Methods

### Ethics statement

The experiment was conducted on private land near the town of Suonenjoki in Central Finland [lat 62° 45.672′, lon 27° 6.015′; ETRS89 geographic coordinates (∼WGS84)]. Permissions for carrying out experiments at this location were obtained from the land owner, whose contact information is available from the authors upon request. The study did not involve endangered or protected species. The experiment was approved by the Finnish Animal Ethics Council (permit ESAVI/1437/04.10.03/2011). Field technicians were trained prior to the experiment and took all possibly precautions to minimise animal stress during trapping and sampling.

### Enclosures and experimental design

The experiment was conducted in 18 adjoining field enclosures (20×25 m each) with natural meadow vegetation, dominated by the grasses *Phleum pratense* and *Deschampsia caespitosa*. Enclosures were constructed of sheet metal rising approximately one meter above ground and extending 50 cm underground. Thereby, vole movement between enclosures was prevented and access by mammalian predators of voles (mustelids) restricted. Avian predators had access to the enclosures but were very rarely observed in the area during the experiment. Each enclosure contained eight sheet-metal shelter boxes (40×40×50 cm, with two entrance hole at the base) approximately 10 m apart, in a 3×2×3 configuration. An Ugglan Special live trap (Grahnab, Sweden) was placed in each shelter box.

Enclosures were randomly allocated to one of three treatment groups: 1) *ad libitum* high-protein (30 per cent dry weight crude protein) food supplementation, 2) *ad libitum* low-protein (1 per cent) food supplementation, or 3) control (no food supplementation). The energy content of the two protein treatments (30% and 1%) was unchanged at 3500 kcal/kg. Food supplementation was supplied through specifically formulated pellets (Altromin, Lage, Germany) available from a wire mesh feeder placed in each shelter box.

At the beginning of June 2011, six field voles (three males, three females) were introduced to each enclosure. The first trapping occasion was conducted two weeks later to obtain baseline abundance estimates representative of established individuals. A total of two male and three female voles were introduced to four enclosures (2 high protein and 2 low protein) to replace voles that had apparently died between introduction and baseline trapping. Food supplementation began immediately following baseline trapping on June 16, 2011, and continued until immediately prior to the final trapping in mid-September (13^th^) 2011. The experiment thus encompassed the primary reproductive period of field vole populations in central Finland [15].

### Vole monitoring and sampling

Abundance monitoring and vole blood sampling was conducted every fourth week for a total of four trapping occasions. On each occasion, traps were baited with oats and checked consecutively at 7 am, 2 pm and 9 pm, for a total of 8–9 times over three days. An electronic PIT-tag (EID Aalten BV, Aalten, Netherlands) was subcutaneously injected into every vole upon first capture and the unique identification number recorded at each encounter. Voles were placed into ventilated buckets and taken to an on-site field laboratory where their sex and reproductive status (males: subadult, mature; females: subadult, mature, pregnant and/or lactating) was determined through external examination. Body mass and head width were measured (to nearest 0.1 g and 0.1 mm, respectively), and approximately 150 µl of blood was collected from the retro-orbital sinus with heparinized capillary tubes. Blood was not collected from juvenile individuals weighing under 15 g. Voles were then released into the same enclosure as captured, except on the final trapping occasion when voles were removed from enclosures. Upon encountering an individual that had already been sampled for blood during the trapping occasion, the vole was immediately released at the point of capture after recording its identification number, sex, reproductive status and weight.

Vole abundance (hereafter density) was estimated separately for each enclosure and trapping occasion (18 enclosures × 4 occasions  =  72 population density estimates) using the program CAPTURE [42]. M_h_ models (which incorporate heterogeneity in capture rates) with the jackknife estimator were employed for trapping occasions one to three. Throughout this period, four enclosures experienced one trapping occasion in which no individual was recaptured after their initial capture. In these cases, density was estimated with removal (M_bh_) models (Pollock and Otto's estimator [43]). During the final trapping occasion, voles were removed from enclosures upon first capture and density was estimated with removal models. Rarely, voles were found dead in traps or died during sampling (approximately 3% of captures). These individuals were excluded from the density estimation models, but added to the final estimate [42]. A population growth rate was calculated for each trapping interval based on the formula, R_t_  =  ln(N_t-1_/N_t_), where N_t_ is the population density at time t [18], [44].

Vole survival rate was calculated separately for each enclosure and trapping interval using program MARK 7.0 [45]. Since survival estimates partially depend on recapture rate, Akaike's information criterion (AIC) -based model selection was employed [46] to compare recapture rate models including enclosure, trapping occasion, their permutations or only the intercept. Due to a small difference in AIC values between the two most parsimonious models (ΔAIC <2), final survival estimates were obtained using a weighted model averaging procedure, taking model selection uncertainty into account [46].

### Condition indices

Body condition index was expressed as the studentized residuals of a random coefficients regression model of individual body mass on head width [47]. Identity of the head width measurer was entered as a random factor in the model to adjust for potential individual variation in head width measurements. Only mature males were included in the analysis of condition index to avoid confoundment by juveniles and reproducing females.

Vole blood was centrifuged at 12 000 g for five minutes, and haematocrit expressed as the percentage of packed red blood cells in total volume. Blood plasma was then separated and frozen (<−20°C) before enzyme-linked immunosorbent assays (hereafter, ELISA).

Total IgG antibody titres were measured according to the following protocol. Solid anti-mouse conjugate was pushed through a 0.22 µm syringe filter and dissolved in 0.135 M NaCl. Plate wells were then coated with 50 µl of anti-mouse IgG (M-8642, Sigma, lot 060M6082) solution (1 mg/ml) and incubated for a minimum of 12 hours at +4°C. Wells were emptied, and masked with 100 µl 1% bovine serum albumin in phosphate-buffered saline (BSA-PBS) and incubated for 60 minutes at room temperature. Wells were then emptied, washed and pat dried. 50 µl of plasma sample (diluted at 1∶40000 with BSA) was added to duplicate wells. A standard was prepared by combining 2 µl from each sample over all trapping occasions. Duplicate standard concentrations of 200, 150, 100, 50, 25, 10, 5 and 0 were run on each plate simultaneously with samples. Plates containing samples and standards were then incubated for 3 hours at room temperature. Following incubation, solutions were removed and the wells washed. 50 µl of alkaline phosphatase conjugated anti-mouse IgG (A-2179, Sigma, lot 31K4852), diluted at 1∶4000 with BSA-PBS, was added to each well and plates incubated for a minimum of 12 hours at +4°C. Following incubation, wells were washed and pat dried, and 50 µl of substrate (1 mg pNPP [P4744, Sigma, lot 109K6076] to 1 ml DEA buffer) was added to each. Plates were then incubated in the dark and read at 405 nm with a Thermo Labsystems Multiskan Ascent 354 platereader after 15, 30, 45, 60 and 75 minutes. An absorbance approximately mid-way between the standard dilutions is most desirable. After comparing absorbance levels, 45 minutes of incubation was deemed the most appropriate. The mean absorbance of the sample duplicates was used as the final measure. On rare occasions when an anomalous result occurred, the plausible duplicate was used alone.

A commercially available mouse-albumin ELISA kit (Alpha Diagnostics International, Texas) was used to measure the albumin concentration of vole plasma as per manufacturer's instructions. An anti-mouse albumin-HPR conjugate was used and plates were read at 450 nm using the Thermo Labsystems Multiskan Ascent 354 platereader.

### Statistical analyses

Random coefficients regression models (PROC MIXED) were used to evaluate the individual and interactive effects of time (week of year as a continuous variable) and treatment on vole density, with the intercept and week as random effects. The effect of population mean condition index on density was evaluated in a separate model. For this, data were restricted to the final three trapping occasions, and condition at the previous trapping occasion (t-1) set as an initial explanatory variable, along with time, treatment and their interactions. The intercept and time were again used as random effects.

Due to the positive correlation between density and week (P<0.001), analyses of growth rate (R_t_), survival, condition index, total IgG, haematocrit and albumin content were carried out with repeated-measures mixed ANOVA models (PROC MIXED) with trapping occasion as a repeated categorical variable. Other initial fixed explanatory variables were treatment, density and all possible interactions. Enclosure and enclosure × trapping occasion were included as random factors (for IgG and albumin models the ELISA plate number was also included as a random factor). Repeated covariate type (autoregressive, unstructured, compound symmetry or toeplitz) selection was based on AIC of the full model. Model selection was thereafter based on a stepwise reduction approach, guided by AIC values and biological importance, using Kenward and Roger estimation [48]. Model comparisons were made using the maximum likelihood (ML) method, and final values obtained from the most parsimonious model with restricted maximum likelihood (REML). Sexes were analysed separately when possible, and model validity was verified via the residual distribution. To assess for delayed effects of density, the data were restricted to the final two trapping occasions and each response model incorporating current density compared to models including densities for the two preceding trapping occasions (t-1, t-2). Unstructured repeated covariate type was employed in these reduced models.

To facilitate interpretation of a three-way interaction between density, treatment and trapping occasion in the final survival model (Table 1), a mixed model was constructed with density to explain survival. Enclosure, with intercept, was set as a random factor. Residuals of this model were then used as the response variable in a repeated ANOVA model in which survival was explained by treatment, trapping occasion and their interaction, as per the methods described above.

**Table 1:** Most parsimonious model to explain each response variable.

Response	Source of variation	Num. df	Denom. df	*F*	*P*
Density	week	1	56	45.35	<0.0001
	treatment	2	51	6.35	0.0034
	week × treatment	2	56	8.12	0.0008
Growth rate	occasion	2	22	3.08	0.07
	treatment	2	11	4.22	0.0445
	density	1	32	11.12	0.0022
Survival	occasion	2	32	6.55	0.0042
	treatment	2	35	4.98	0.0126
	density	1	36	4.71	0.0368
	density × treatment	2	35	4.60	0.0168
	density × occasion	2	34	7.20	0.0025
	treatment × occasion	4	31	1.67	0.18
	density × treatment × occasion	4	33	2.76	0.0434
Prop. males	occasion	3	44	2.53	0.07
	treatment	2	119	0.41	0.67
	density	1	119	1.41	0.24
	treatment × occasion	6	119	0.89	0.50
Prop. reproducing females	occasion	2	26	2.94	0.07
	treatment	2	51	0.78	0.47
	density	1	51	7.12	0.0102
	treatment × occasion	4	51	1.04	0.40
Prop. <20 g	occasion	2	227	3.06	0.06
	treatment	2	79	0.13	0.89
	density	1	79	7.62	0.0072
	density × occasion	2	79	3.36	0.0397
	treatment × occasion	4	79	5.27	0.0008

Final values were obtained with REML. Full models contained time (week or trapping occasion), treatment group, density, and all their interactions as initial explanatory variables. Trapping occasion is a categorical variable. Week denotes the week of year and is continuous. Enclosure and enclosure × time were set as random variables.

Generalized linear mixed models (PROC GLIMMIX), employing the same methodology and fixed and random factors, were used to evaluate changes in the proportion of males (sex ratio), voles weighing less than 20 g (as representative of juvenile recruitment), and reproducing females from the total female population. As external signs of reproduction or juveniles were not yet present at the onset of the experiment, baseline data were removed from these models. Generalized models were assessed for over-dispersion. Data were analysed in SAS version 9.3 (SAS Institute Inc., Cary, NC).

## Results

### Population size

**Figure 1:**
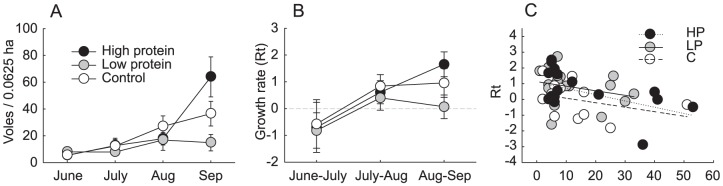
Size and growth of experimental field vole populations. (A) density (mean ± se), (B) growth rate (Rt) (least squared mean ± se), (C) population growth rate by density.

The effect of treatment on density changed over time (Table 1). Densities were similar among treatment groups for the initial three trapping occasions (Figure 1a). However, by September densities were greater in high protein than low protein treatment populations, while control groups displayed an intermediate level of density, which did not differ from either supplementation group. Population density was not influenced by mean condition index (F^condition index (t-1)^_1, 38_  =  2.47, P = 0.124).

Mean population growth rates were predominantly positive throughout the experiment and varied between treatment groups (Table 1, Figure 1b). Population growth rate was negatively associated with density in all treatment groups (Table 1, Figure 1c).

### Demographics and survival

**Figure 2:**
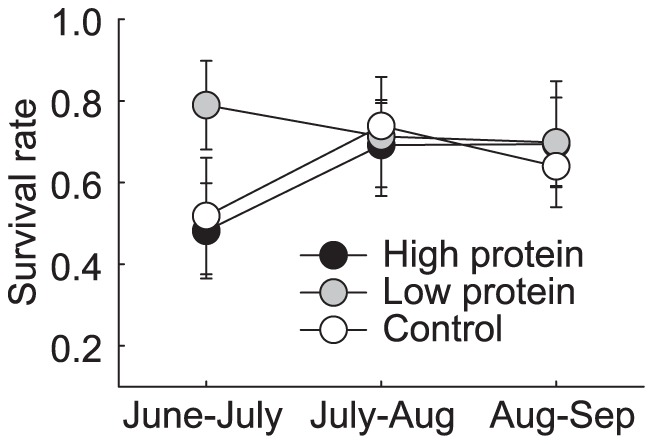
Treatment-wise population survival rate (mean ± se).

Survival rates differed with density, treatment and time (Table 1). Survival was higher in low protein populations than in other groups from June to July (Figure 2). From July to September, all rates stabilized with approximately 70% of voles surviving between trapping occasions (Table 1). Neither treatment (F^treatment^_2, 18_  =  0.80, P = 0.47) nor trapping occasion (F^occasion^_2, 33_  =  0.96, P = 0.39) affected survival in the density-corrected model.

**Figure 3:**
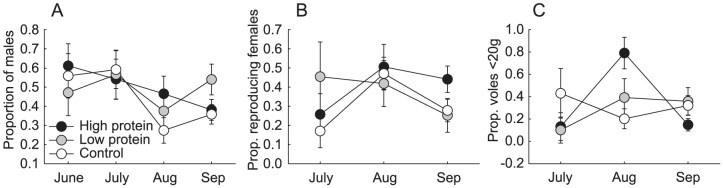
Demographic attributes of experimental populations (least squared mean ± se). (A) proportion of males in total population, (B) proportion of reproducing (pregnant and/or lactating) females in total female population, (C) proportion of voles <20 g from total population.

The sex ratio of the populations was unaffected by the treatments (Table 1, Figure 3a). Meanwhile, the proportion of reproducing females, out of all females, decreased with increasing population density, regardless of treatment (Estimate  =  −0.0161, s.e  = 0.006, Table 1, Figure 3b). The proportion of juvenile voles (<20 g) varied between treatment groups and trapping occasions (Table 1, Figure 3c), being highest in control populations in July, and lowest by August. High protein populations displayed the greatest proportion of juveniles in August, but lowest in September.

### Indicators of condition

**Figure 4:**
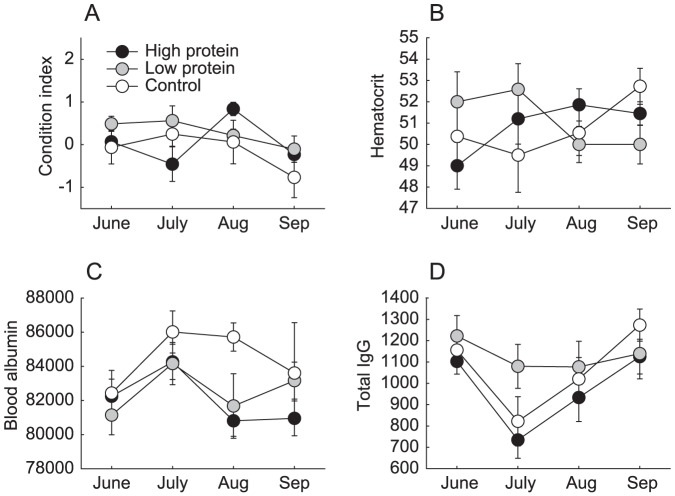
Condition indices of male voles from experimental populations (mean ± se). (A) body condition index, (B) haematocrit, (C) plasma albumin, (D) total IgG antibody titer.

No associations between treatment and body condition index were identified (Figure 4a). Nor were there significant differences between treatment groups in male haematocrit (Figure 4b). Male haematocrit was negatively density dependent at the beginning of the experiment, but the relationship dissipated with time (F^density×occasion^_3, 62_  = 4.35, P = 0.008). Density in the previous trapping occasion explained male haematocrit from August to September better than current density (ΔAIC  = 2.0). Male haematocrit thus exhibited delayed density dependence in high protein populations (F^density(t-1)×treatment^_2,17_  = 4.22, P = 0.032). A negative effect of density on male albumin, that was present at the beginning of the experiment, relaxed with time (F^density×occasion^_3, 86_  =  2.62, P = 0.056, Figure 4c). Current density explained male albumin levels better than past density (ΔAIC  = 4.7), but none of the explanatory variables reached significance. Meanwhile, male IgG was higher in June (t = 2.69, d.f  = 5, P = 0.046) and September (t = 3.55, d.f = 12, P = 0.004) than July, but did not vary between treatment groups (F^occasion^_3, 8_  = 5.05, P = 0.032, Figure 4d). Density in the previous trapping occasion was again a better predictor of male IgG than current density (ΔAIC  = 3.2), but none of the explanatory variables reached significance.

**Figure 5:**
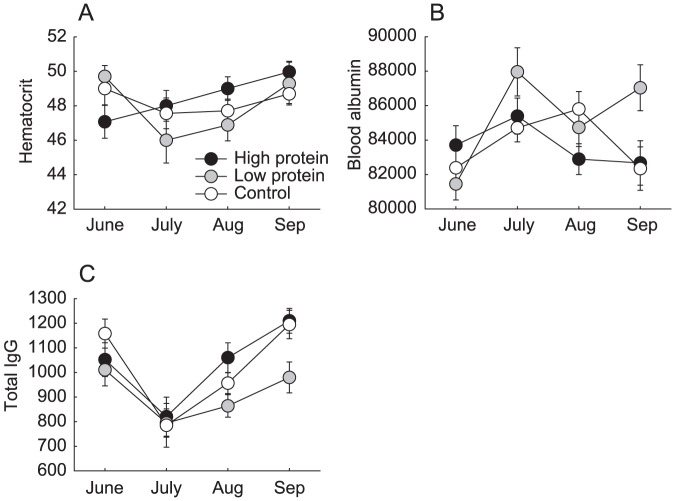
Condition indices of female voles from experimental populations (mean ± se). (A) haematocrit, (B) plasma albumin, (C) total IgG antibody titer.

Female haematocrit consistently increased in high protein populations during the experiment (Figure 5a). However, interpretation is confounded by three-way interactions with both current and past density (F^density×occasion×treatment^_6, 34_  = 33.8, P = 0.039; F^density(t-2)×occasion×treatment^_2, 77_  = 3.18, P = 0.047). Meanwhile, no significant effects on female albumin were identified (Figure 5b), including density two occasions prior, which explained the data better than current density (ΔAIC  = 6.6). No effects of treatment group were identified in female total IgG in the full model (Figure 5c). However, a delayed density-dependent decrease in female IgG present in August, had disappeared by September (F^density(t-1)×occasion^_1, 93_  = 7.56, P = 0.007).

## Discussion

Consistent with our hypothesis, the summer growth of vole populations was limited by the availability of dietary proteins. Food resources are of great importance to the population dynamics of herbivores [49]–[51], including cyclic small mammals [17], [52]–[53]. In general, the quantitative effects of resources on vertebrate herbivore populations have been extensively studied [54], while the effects of food resource quality remain little investigated. Similarly, in voles the limiting effects of food on population demography has manifested through quantity, and predominately only during winter [18]. As such, our experimental results offer important insights into processes contributing to variation in herbivore density – namely the availability of high-quality food during the growing season.

Survival rates of voles did not differ between treatment groups during the experiment. Therefore, the observed differences in density are largely attributable to increased recruitment through reproduction. This is supported by the tendency of high protein populations to consist of few males and many reproducing females as compared to the other treatment groups. However, increased rates of reproduction were not reflected in the proportion of juvenile voles, which were lowest in September when the population growth rate was highest. Desy and Batzli [12] identified the same peculiarity which they attributed to faster growth of juveniles with food supplementation. In other words, high quality food enables voles to grow faster than lower quality food. Indeed, protein supplementation has been found to accelerate the growth of small rodent individuals [55]. For this reason, it was not appropriate to evaluate functional group differences in survival and condition between juvenile and adult voles in the current experiment. It should be noted that the quality of available food resources may affect the demographic rates of vole populations differently in winter than in summer – this remains a topic for further experimentation.

Interestingly, control treatment groups in our experiment attained approximately half the densities of high protein supplemented groups (Fig. 1). The protein content of our high-quality supplementation was 30%, while crude protein levels in grasses (including *Phleum pratense*) at the end of the growing season are about 10–15% of dry weight [56]. It is therefore tempting to entertain the idea that summer vole densities closely reflect the levels of dietary protein available to voles in their forage.

Contrary to our predictions, neither body condition nor haematological indices were clearly associated with experimental treatments or population density. Nevertheless, the identification of changes over time, and interactions with current and past density, highlight the complex interactions and potential utility of these measures in population ecology research. It should be noted that interpretation of haematological indices is difficult and several parameters are usually required to provide an adequate representation of health status [57]. For example, elevated total IgG could represent high baseline immunity levels resulting from good health or an immune response to infection [39]. Similarly, low albumin may be a sign of a protein deficient diet or infection [58]. Since we employed several health indicators without observing treatment effects, it appears that voles were able to maintain good physiological condition during the breeding season on natural food resources alone (for contrasting results during the non-breeding season, see [39]). In the context of our treatments, it seems plausible that voles which received supplemental protein were allocating it foremost to reproduction, as opposed to elevating their own physiological condition (i.e., income breeding [59]).

Korpela et al. recently highlighted the association between summer growing conditions and the dynamics of vole populations [19]. However, their study did not identify proximate mechanisms acting during summer. Climate, amongst other things, has been shown to alter nitrogen levels in plants [31]–[32], and we have demonstrated here that summer protein levels are a plausible mechanistic link between climate and vole population demography. In further support, a ‘midsummer crisis’, hypothesized to result from a shortage of high-quality food due to graminoid senescence [15], did not present in high protein treatment groups. Meanwhile, the growth rate of low protein populations was clearly reduced between the final two trapping intervals without obvious changes in survival rates. Further research is nonetheless needed to elucidate causalities between climate and herbivore diet quality.

Considerable debate has focused on factors which limit and regulate cyclic populations of small mammals. A common line of differentiation is between intrinsic (for example age structure and maternal or juvenile environment: see [60]–[63]) and extrinsic factors (the environment, including predation: see [6], [9]. However, recent transplant experiments have provided compelling evidence to support an important effect of the immediate environment on the life history traits of voles [64]–[65] (see however [63]). Our identification of diet quality limitation on increasing vole populations is consistent with the latter findings. Specifically, we have demonstrated that protein availability limits the growth of summer vole populations. We therefore conclude that diet quality, ultimately determined by stochastic variation in climate, is likely to have a hitherto underestimated influence on the population dynamics of small mammals.
